# Exploring the association between social and emotional skills and online social experiences: a network analysis and latent profile analysis

**DOI:** 10.3389/fpsyt.2025.1582272

**Published:** 2025-04-30

**Authors:** Xiaoli Yang, Junyu Yan, Ziqian Cui

**Affiliations:** ^1^ School of Economics and Management, Yanshan University, Qinhuangdao, China; ^2^ Department of Educational Psychology, East China Normal University, Shanghai, China; ^3^ School of Psychology, South China Normal University, Guangzhou, China

**Keywords:** online social experiences, socio-emotional skills, stress resistance, network analysis, latent profile analysis

## Abstract

With the widespread use of social media, online social experiences have become increasingly influential on the mental health of young people. However, how individuals’ socio-emotional skills modify these experiences remains unclear. This study conducted a survey of 1,422 Chinese university students and applied network analysis and latent profile analysis to explore the relationship between socio-emotional skills and online social experiences. The results showed that socio-emotional skills had a significant positive correlation with positive online social experiences and a significant negative correlation with negative online social experiences. Among these skills, stress resilience may serve as a key dimension for enhancing overall socio-emotional competence. Therefore, interventions targeting stress resilience could be particularly effective in helping individuals strengthen their broader socio-emotional skills, thereby improving their online social experiences. Additionally, the study found that positive and negative online social experiences are two distinct constructs, suggesting that intervention strategies should address them separately. These findings provide a theoretical foundation for developing targeted interventions to improve young people’s online social experiences and promote their mental well-being.

## 1 Introduction

The rapid advancement of the Internet and digital technologies has led to the widespread use of social media. According to recent statistics, as of 2024, over 5 billion people worldwide use social media platforms (1). Young people aged 18–29 constitute the primary demographic of social media users and engage with platforms such as Facebook, WeChat, and TikTok almost daily to share information, express opinions, and communicate with others (2). In contrast to traditional media, social media offers an interactive platform that enables young people to present themselves and reach a broader audience (3). This interactivity facilitates the formation of online social relationships and strengthens interpersonal connections.

Unlike face-to-face offline interactions, online interactions via social media break through the barriers of time and space, expanding the size of social networks and increasing the potential for obtaining social support (4). Meanwhile, the unique characteristics of online interactions, such as asynchronicity and anonymity (5), can lead to more superficial social relationships and increase the risk of being judged and encountering threats (6, 7). Thus, the effects of social media depend on the quality of the social interactions it facilitates. Based on the interpersonal-connection-behaviors framework (8), when social media promotes meaningful social connections, it can lead to a positive online experience. However, if these interactions fail to fulfill individuals’ sense of belonging, they may result in negative online experiences.

Positive online social experiences, defined as online interactions that foster social connections through emotional, informational, belonging, and capitalization support (9), are widely recognized as a supplement to offline social support. For individuals who struggle to obtain offline social support, positive online social experiences can serve as a vital source of security. For example, sexual and gender minority youth mitigate their feelings of depression through positive online social experiences (10), while people with disabilities gain the opportunity to enhance their self-efficacy (11). The effects of positive online social experiences were more pronounced during the COVID-19 lockdown period, significantly alleviating the loneliness of people under home quarantine (12). While the benefits of positive online social experiences are well-documented for marginalized groups, research remains relatively scarce for more typical populations and everyday situations. One exception is Cole et al. (13), who found that positive online social experiences have a significant impact on the physical and mental health of ordinary college students. Uchino et al. (14) showed that as positive online social experiences increase, individuals’ self-rated health tends to improve correspondingly. Shi et al. (15) found that individuals who experience more positive online interactions tend to exhibit higher levels of well-being in real life. Altogether, positive online social experiences have potential benefits for individuals.

While positive online social experiences can provide meaningful social support for groups who are not accustomed to face-to-face offline interactions, they also create opportunities for harmful interpersonal interactions that may undermine mental health. Negative online social experiences, such as stigmatization, online harassment, and cyberbullying (9), have been shown to increase feelings of loneliness, anxiety, and depression, reducing overall sense of belonging and well-being (16, 17), and even lead to the development of suicidal thoughts (18). Moreover, young people who experience negative online interactions may adopt maladaptive coping strategies to manage their psychological distress. For instance, they may engage in cyberbullying behaviors themselves, thereby extending the negative impact to others (19). Existing research has predominantly examined specific negative online experiences, such as cyberbullying, stigmatization, and harassment. However, less emphasis has been placed on the more subtle yet pervasive negative experiences of everyday online interactions, such as social rejection or digital embarrassment. Bonsaksen et al. (20) discovered that these types of experiences can also have adverse effects on mental health. Additionally, Garcia et al. (21) indicated that negative online social experiences correlate not only with poorer sleep quality but also with an increased risk of comorbidity between poor mental and sleep health. In summary, negative online experiences can have detrimental effects on both physical and mental health.

Both positive and negative online social experiences have been shown to significantly influence individuals’ health and well-being (22). As such, understanding the factors that contribute to these experiences is essential, as it can inform the development of targeted interventions. According to the uses and gratifications theory (23), individuals select media content based on their personal needs and motives, and they derive specific gratifications from their media use, highlighting the role of individual differences in modifying media experiences. In the context of online social interaction, individuals are not passive recipients of digital content. Instead, their personal characteristics play a crucial role in determining how they engage with others online and what kinds of experiences they derive from these interactions. As such, personality traits are increasingly recognized as important factors in online social experiences (24). Some studies have investigated online social experiences based on the Big Five personality traits (25–28). For instance, individuals high in extraversion are more likely to actively engage in online interactions, exhibit greater social confidence, and report more positive online social experiences (27). Conversely, those high in neuroticism tend to be more emotionally reactive to negative online content, experiencing heightened social comparison and jealousy, which in turn may contribute to negative experiences (28). In addition, individuals with higher levels of conscientiousness may hold more critical attitudes toward social media, viewing it as unproductive or distracting. This may lead to more negative perceptions of online interactions (25). These findings collectively suggest that personality has been widely recognized as the key factor of online social experiences. Consequently, interventions tailored to personality may offer a promising approach to improving online interpersonal experiences and promoting well-being.

Personality traits are generally considered relatively stable patterns of thinking, feeling, and behaving across time and contexts (29), and are typically less amenable to short-term interventions (30). In contrast, socio-emotional skills emphasize how individuals adapt their thoughts, emotions, and behaviors to meet the demands of specific situations (31). For example, a usually quiet and shy person may still be able to confidently deliver a presentation when needed, demonstrating their socio-emotional skill in adjusting to social situations (32). Although a wide range of socio-emotional skills have been identified, these skills can be conceptually organized into five domains that correspond to the Big Five personality traits (33). This alignment provides a comprehensive and theoretically grounded approach for assessing and categorizing socio-emotional competencies (34). However, unlike the relatively stable nature of personality traits, socio-emotional skills are considered malleable competencies that can be developed and enhanced through learning (35). This makes socio-emotional skills particularly valuable in interventions. In digital environments, where people often face complex and emotional interactions, focusing on socio-emotional skills provides a more practical and adaptable way to understand and improve online social experiences.

In previous research, the impact of online social experiences on individuals’ health and well-being has been widely explored, and studies have also examined the relationship between personality traits and online social experiences. However, from an intervention perspective, personality traits are generally considered stable and difficult to change through short-term interventions. In contrast, socio-emotional skills, being more malleable, offer a more feasible entry point for interventions. Therefore, the aim of this study is to explore the association between socio-emotional skills and online social experiences, identify the key skills that modify online social experiences, and provide support for developing effective intervention strategies to enhance individuals’ online social experiences and overall well-being. This study used network analysis to explore the relationship between socio-emotional skills and online social experiences and identified key variables through centrality measures (36). Interventions targeting these key nodes may more effectively influence the weight and distribution of other nodes within the network. Given that socio-emotional skills are widely recognized as crucial for individuals’ well-being (37), the study hypothesized that socio-emotional skills were positively associated with positive online social experiences and negatively associated with negative online social experiences. Additionally, considering individual heterogeneity, the study further employed latent profile analysis (LPA) to examine whether key dimensions of socio-emotional skills differed across various groups (38), providing support for the development of intervention strategies and the identification of at-risk populations.

## 2 Methods

### 2.1 Participants

The appropriate sample size for the network analysis was determined using the *powerly* package in R (39). Based on the analysis, a sample of 1,367 participants was required to achieve acceptable statistical power (1−β = 0.8, sensitivity = 0.6) for a cross-sectional network model consisting of 17 nodes with a density of 0.4.

Participants were recruited online through convenience sampling by posting an online questionnaire on social media platforms. Inclusion criteria for the present study required participants to identify as college students, spend at least 180 seconds completing the survey, and pass attention checks (e.g. “Select ‘Disagree’ for this question”). After excluding cases with missing data, anomalous responses, and those failing to meet the inclusion criteria, a total of 1,422 Chinese university students were included in the final dataset. This sample size satisfied the estimated requirement and ensured sufficient power for the planned analyses. The final sample comprised 524 (36.85%) males and 898 (63.15%) females, with an average age of 20.95 years (*SD* = 2.76, ranging from 17 to 27). The same sample was also used for the latent profile analysis.

### 2.2 Measures

#### 2.2.1 Online social experiences measure

The Chinese version of the Online Social Experiences Measure (OSEM), originally revised by Kent de Grey et al. (9), was adopted in this study. The scale consists of two dimensions: online social positivity and online social negativity, each comprising 8 items, for a total of 16 items. Participants rated their social media experiences using a 5-point Likert scale ranging from 1 (“Very slightly”) to 5 (“Extremely”), with higher scores on each dimension indicating stronger perceptions of positive or negative experiences on social media. The scale demonstrated good reliability, with Cronbach’s α of 0.96 for online social positivity and 0.92 for online social negativity. Additionally, the scale showed acceptable goodness-of-fit of confirmatory factor analysis (CFA) in the current study (χ^2^(100) = 947.38, CFI = 0.96, TLI = 0.95, RMSEA = 0.08, SRMR = 0.04).

#### 2.2.2 Short form of the survey on social and emotional skills

The short form of the Survey on Social and Emotional Skills (SESS-SF), which was developed by Wang and King (34), was used to assess five core socio-emotional competencies, as conceptualized by the OECD. These competencies include task performance (self-control, responsibility, persistence), emotional regulation (stress resistance, optimism, emotional control), collaboration (empathy, trust, cooperation), open-mindedness (tolerance, curiosity, creativity), and engaging with others (sociability, assertiveness, energy). Each competency comprises three dimensions, with three items per dimension, resulting in a total of 45 items. Responses were recorded on a 5-point Likert scale, ranging from 1 (“Strongly disagree”) to 5 (“Strongly agree”). The scale showed strong internal consistency (Cronbach’s α = 0.93), with subscale reliability ranging from 0.72 to 0.88, and demonstrated a good goodness-of-fit of CFA (χ^2^ (840) = 3791.78, CFI = 0.92, TLI = 0.91, RMSEA = 0.05, SRMR = 0.06) in this study.

### 2.3 Software and statistical methods

Data preprocessing, descriptive statistical analysis, and reliability and validity testing were performed using SPSS 26.0. Harman’s single-factor test indicated that the first factor accounted for only 22.22% of the total variance, suggesting no significant common method bias. The goodness-of-fit test assessed model fit using the CFI, TLI, RMSEA, and SRMR. Model fit was considered acceptable if CFI and TLI exceeded 0.90, RMSEA was below 0.08, and SRMR was below 0.06 (40, 41).

Network analysis was conducted in R 4.4.2 using several specialized packages (42). The *networktools* package was used to examine node redundancy, while the *bootnet* package was utilized for network construction and stability testing. The *mgm* package was employed to estimate node predictability, which quantifies the extent to which a variable’s variance can be explained. The *qgraph* package was used for network visualization and centrality calculations, including strength, closeness, and betweenness (43). Strength centrality represented the total weight of all edges connected to a node. Closeness centrality was defined as the inverse of the total shortest path length from a node to all other nodes. Betweenness centrality reflected how often a node appeared on the shortest path between two other nodes.

Latent profile analysis was conducted in Mplus 8.0 to determine the most meaningful and optimal number of latent profiles (44). To identify the most appropriate model, multiple fit indices were examined, including log-likelihood, Akaike Information Criterion (AIC), Bayesian Information Criterion (BIC), sample size-adjusted BIC (aBIC), and entropy. Lower values of AIC, BIC, and aBIC indicate better model fit, while higher entropy values reflect more accurate classification, with values above 0.8 generally signifying that 90% of cases are correctly classified. In addition, likelihood ratio tests, specifically the Lo-Mendell-Rubin (LMR) test and the bootstrapped likelihood ratio test (BLRT), were conducted. These tests compared models with profile *k* and profile *k*–1, where a significant result suggests that the profile *k* model provides a superior fit. Furthermore, this study explored the impact of socio-emotional skills’ latent profiles on online social experiences. The Bolck-Croon-Hagenaars (BCH) method was employed, a model-based approach that estimates differences in distal outcomes across latent profiles while accounting for classification uncertainty (45). In this study, the BCH method was used alongside one-way ANOVA to examine how different latent profiles of socio-emotional skills influence online social experiences.

## 3 Results

### 3.1 Correlation analysis between online social experiences and socio-emotional skills

**Figure 1:**
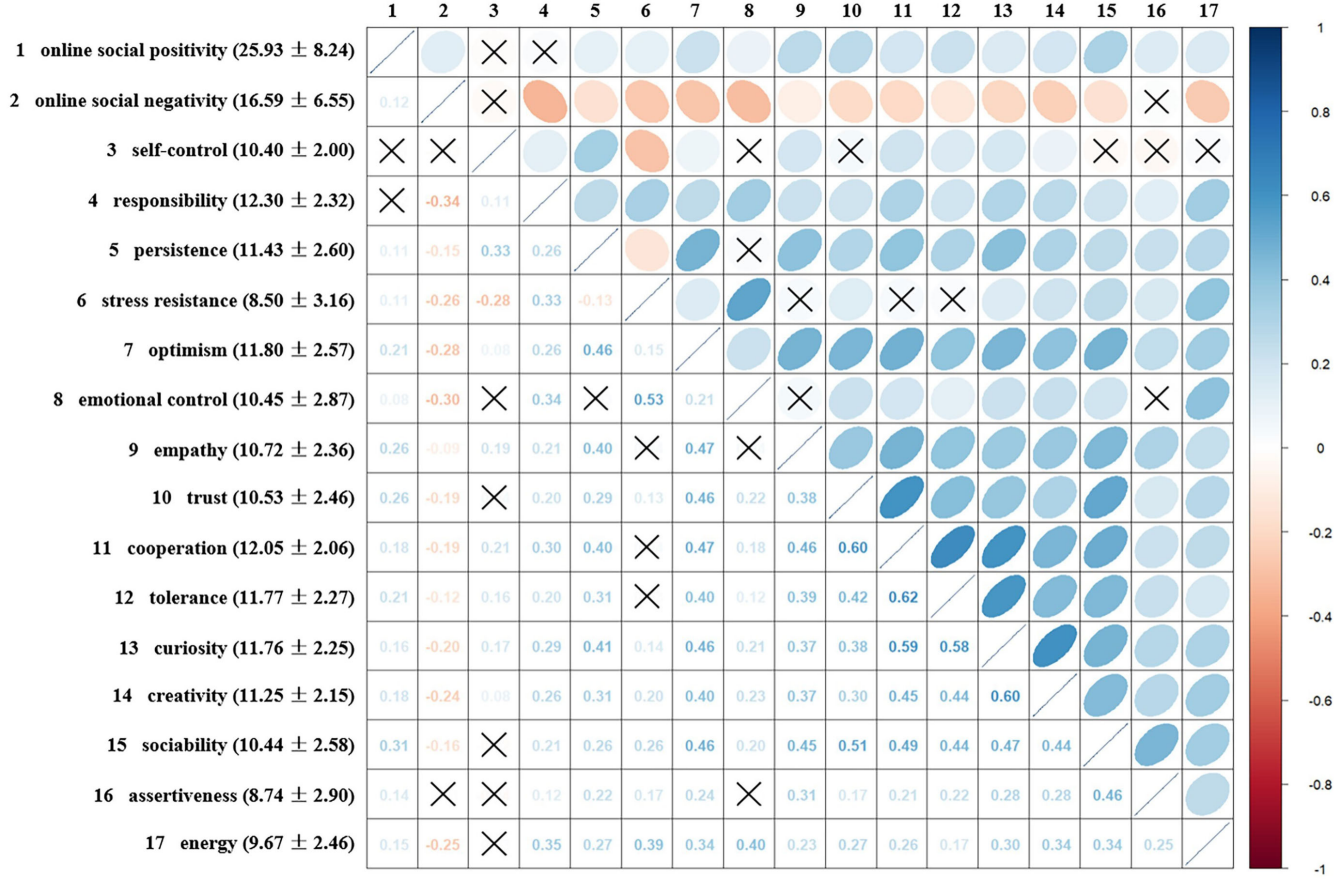
Descriptive statistics and correlation matrix of online social experiences and socio-emotional skills. The color and orientation of the ellipses indicate the direction of the correlations (with blue and a bottom-left to top-right orientation representing positive correlations). The depth of color and the shape of the ellipses reflect the strength of the correlations (darker colors and more elongated shapes indicate stronger correlations). A “×” denotes non-significant correlations with *p* > 0.05.

**Figure 1** presents the descriptive statistics for online social experiences and socio-emotional skills, along with the correlation matrix between these variables. Significant correlations were observed among most variables. Specifically, most sub-dimensions of socio-emotional skills exhibited significant positive correlations with each other (*r* ∈ [0.08, 0.62], *p* < 0.01), except for stress resilience, which showed significant negative correlations with both self-control (*r* = −0.28, *p* < 0.001) and persistence (*r* = −0.13, *p* < 0.001). In addition, the majority of socio-emotional skill sub-dimensions were positively correlated with online social positivity (*r* ∈ [0.08, 0.31], *p* < 0.01) and negatively correlated with online social negativity (*r* ∈ [−0.34, −0.09], *p* < 0.001). Notably, online social positivity and online social negativity were also positively correlated (*r* = 0.12, *p* < 0.001). Further details are provided in **Figure 1**.

### 3.2 Network analysis of online social experiences and socio-emotional skills

**Figure 2:**
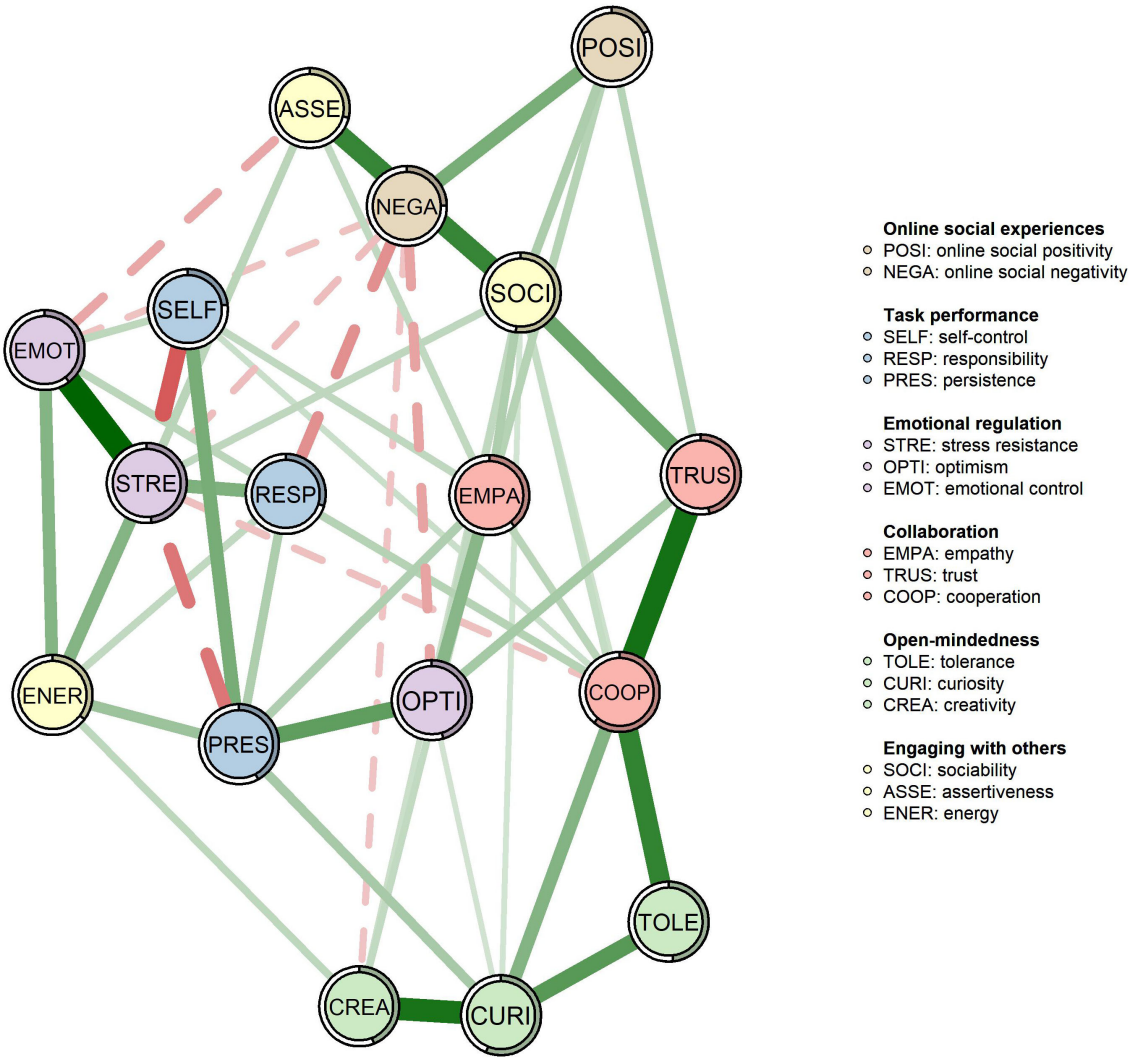
The network analysis of online social experiences and socio-emotional skills. The color and style of the edges in the network indicate the direction of the correlations (with green and solid lines representing positive correlations). The thickness of the edges reflects the strength of the correlations (with thicker edges corresponding to stronger correlations). The circular rings around the nodes represent the variance of the factors represented by the nodes.

To examine the relationships between online social experiences and socio-emotional skills, a network analysis was conducted using the extended Bayesian information criterion with a tuning hyperparameter of 0.5 (36), as shown in **Figure 2**. It is important to note that no variables were excluded due to redundancy, which means each variable exhibited at least 25% statistically significant differences in correlations with all other variables within the network (46).

As shown in **Figure 2**, each node was surrounded by a ring representing the variance of the corresponding variable. The shaded portion of the ring indicates the proportion of the variance explained by its connected variables, also known as predictability. In this network, the predictability of online social negativity was 0.25, meaning that 25% of its variance could be accounted for by its neighboring nodes. Similarly, the predictability of online social positivity was 0.18, suggesting that 18% of its variance was explained by its surrounding nodes. The average node predictability was 0.40, indicating that, on average, 40% of the variance in these nodes could be attributed to their connections within the network model.

The network consisted of 17 nodes and 53 edges, yielding a density of 0.39. Among these edges, 44 represented positive relationships, while 9 reflected negative relationships. The strongest positive and negative edges were both associated with stress resistance, specifically the connection between stress resistance and emotional control (weight = 0.39) and the negative relationship between stress resistance and self-control (weight = −0.25). Most negative edges were linked to online social negativity, with the strongest negative association observed between online social negativity and responsibility (weight = −0.17), followed by its connections to optimism (weight = −0.14), stress resistance (weight = −0.10), emotional control (weight = −0.09), and creativity (weight = −0.09). In contrast, online social positivity was positively associated with empathy (weight = 0.11), trust (weight = 0.11), and sociability (weight = 0.12). It is important to emphasize that the edge between online social positivity and online social negativity was positive (weight = 0.20). Further details are presented in **Figure 2**.

**Figure 3:**
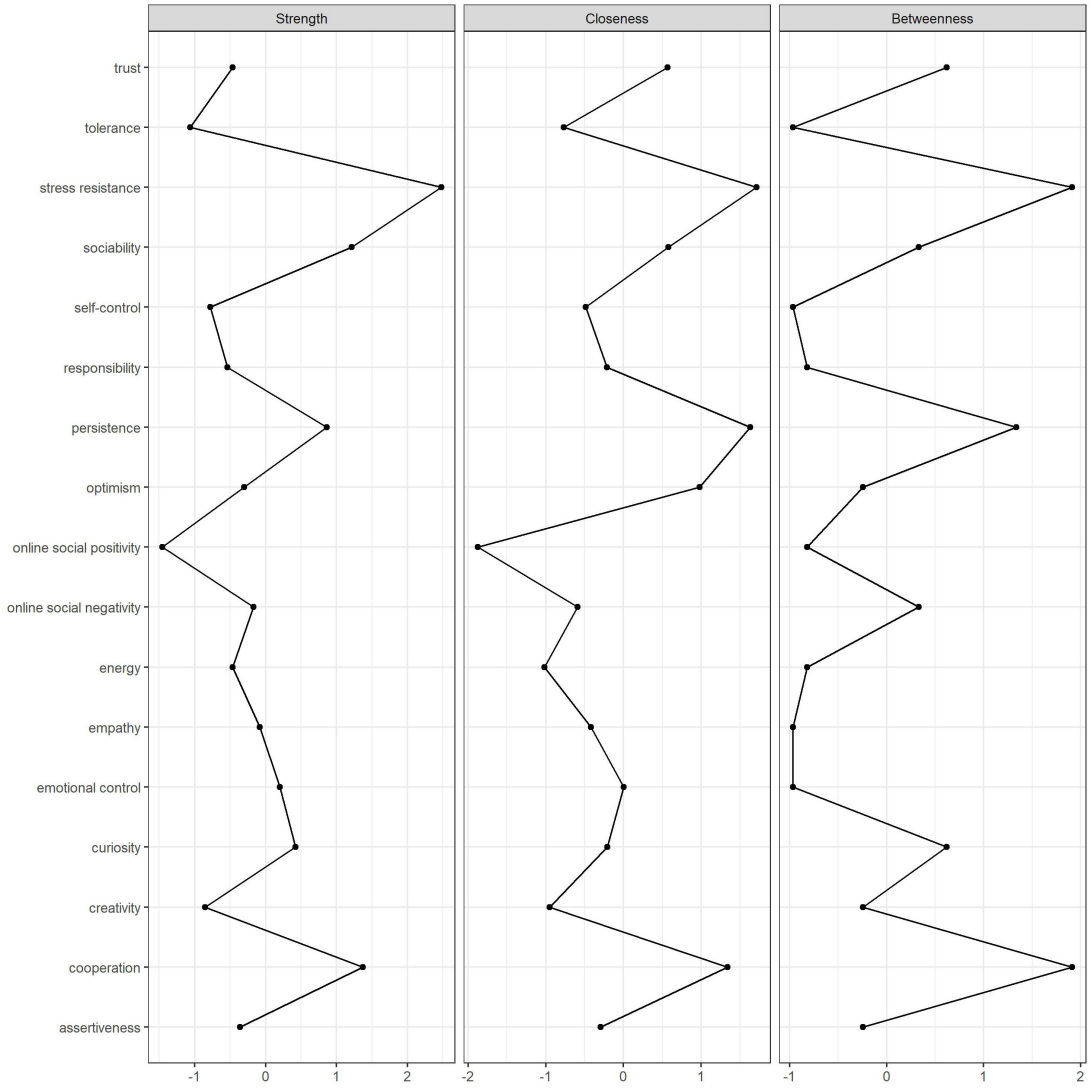
The centrality plot of the network.

**Figure 3** displays the standardized centrality scores for each variable in the network. Stress resistance exhibited the highest strength centrality, closeness centrality, and betweenness centrality, underscoring its critical role and strong influence within the network. Other sub-dimensions of socio-emotional skills, such as persistence and cooperation, also exhibited high centrality, indicating their significant influence on the overall network structure. In terms of online social experiences, online social negativity showed higher centrality indices compared to online social positivity, indicating a stronger influence on other nodes within the network. The correlation stability (CS) coefficients were 0.59 for node strength and 0.36 for both node closeness and betweenness, suggesting that these indices remained correlated with the original data (*r* = 0.7) after removing 59% of the data for node strength and 36% for node closeness and betweenness. Overall, the bootstrapped stability of the centrality analysis confirmed that the network demonstrated good stability.

### 3.3 Latent profile analysis of socio-emotional skills

A latent profile model was constructed using the scores of 15 sub-dimensions of socio-emotional skills as observed variables. Beginning with an initial model, the number of profiles was gradually increased, resulting in five latent profile models for comparison. The fit indices for different latent profile models are presented in **Table 1**. As the number of profiles increased, the log-likelihood, AIC, BIC, and aBIC values showed a decreasing trend. Model selection based on entropy indicated that the four-profile model provided the best fit. However, the LMR test for the four-profile model was not significant, suggesting that the three-profile model offered a better fit. Moreover, the smallest class in the four-profile model accounted for less than 5% of the total sample. Considering these factors, the three-profile model was ultimately selected as the best-fitting model.

**Table 1:** The indexes of model fit.

Model classes	Log likelihood	AIC	BIC	aBIC	Entropy	LMR(*p*)	BLRT(*p*)	Profile probability
1	-49346.22	98752.44	98910.24	98814.94	–	–	–	1
2	-47593.29	95278.58	95520.53	95374.40	0.81	0.01	<0.001	0.60/0.40
3	-46802.80	93729.60	94055.71	93858.76	0.87	<0.001	<0.001	0.24/0.57/0.19
4	-46388.73	92933.45	93343.72	93095.94	0.89	0.17	<0.001	0.54/0.01/0.27/0.18
5	-46197.25	92582.51	93076.93	92778.33	0.85	0.01	<0.001	0.01/0.27/0.14/0.41/0.17

AIC, Akaike information criterion; BIC, Bayesian information criterion; aBIC, sample size-adjusted BIC; LMR, Lo–Mendell–Rubin; BLRT, bootstrapped likelihood ratio test.

**Figure 4:**
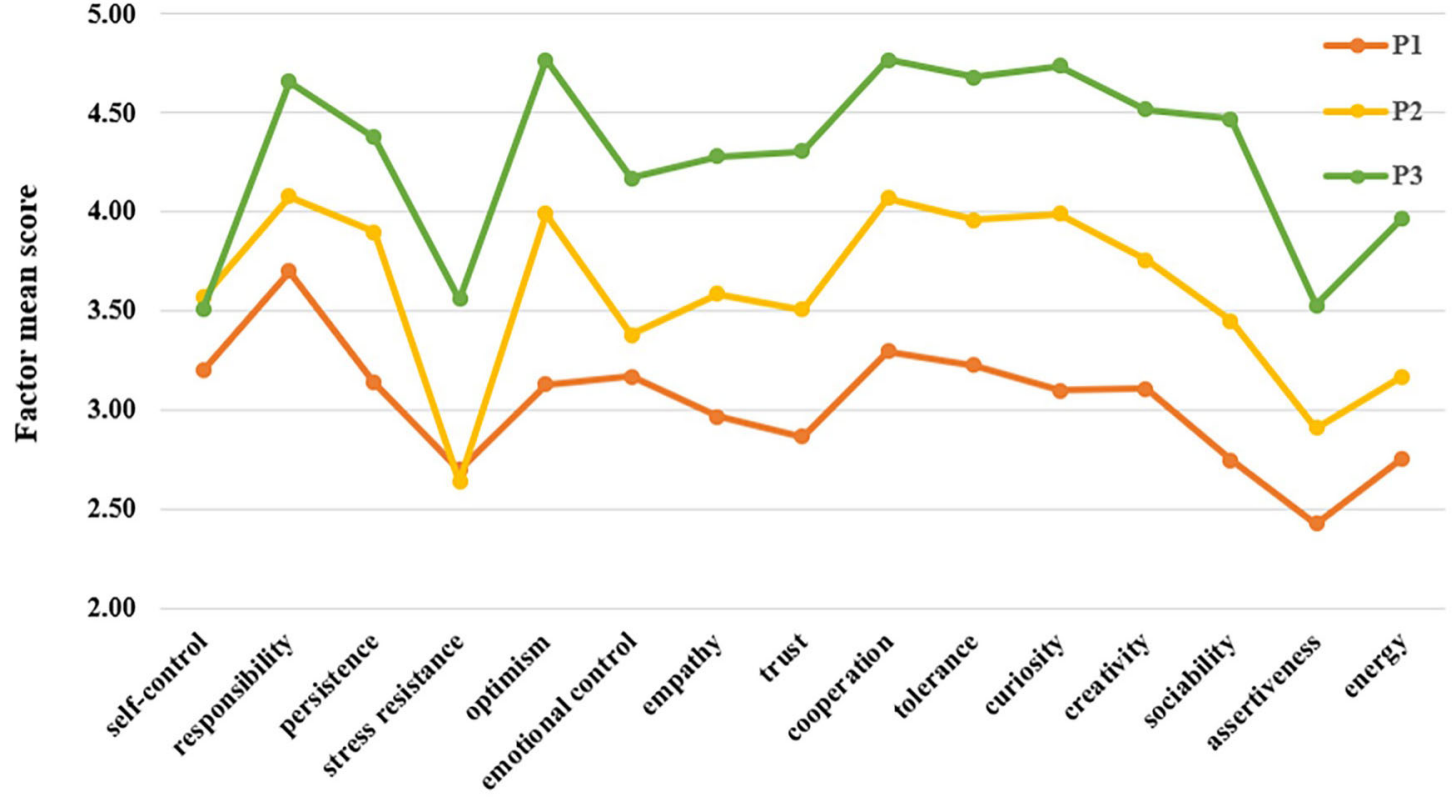
Average scores of socio-emotional skills sub-dimensions across three profiles.

**Figure 4** illustrates the mean scores of each socio-emotional skills sub-dimension across the three identified profiles. Profile 1 comprised 339 participants (23.84%) who demonstrated relatively low scores across all sub-dimensions of socio-emotional skills. Consequently, this profile was labeled the “low socio-emotional skills group” or, for brevity, the “low type.” Profile 2 included 812 participants (57.10%) whose scores were generally around the average across all sub-dimensions, except for a lower score in stress resistance. This group was designated as the “moderate socio-emotional skills group” or the “moderate type.” Profile 3 consisted of 271 participants (19.06%) who scored higher than the other two profiles in most dimensions, except for self-control, which was comparatively lower. This profile was identified as the “high socio-emotional skills group” or the “high type.”

Further analysis using a one-way ANOVA, as shown in **Table 2**, revealed significant differences across the three profiles in all sub-dimensions of socio-emotional skills. *Post hoc* tests indicated that the three profiles exhibited significant differences in all sub-dimensions (*p* < 0.001), except for self-control and stress resistance. In most dimensions, the scores followed a consistent pattern (low type < moderate type < high type). However, for self-control, the low type scored significantly lower than both moderate and high types (*p* < 0.001), with no significant difference observed between the moderate and high types (*p* = 0.51). Similarly, in the stress resistance dimension, the high type scored significantly higher than low and moderate types (*p* < 0.001), whereas no significant difference was found between the low and moderate types (*p* = 0.60).

**Table 2:** The differences on the socio-emotional skills sub-dimensions across three latent profiles.

	Socio-emotional skills type (*M* ± *SD*)				
Variable	P1 (*n* = 339)	P2 (*n* = 812)	P3 (*n* = 271)	*F*	*Post hoc* tests
self-control	9.59 ± 1.96	10.70 ± 1.83	10.53 ± 2.27	39.58^***^	P1 < P2 = P3
responsibility	11.09 ± 2.44	12.24 ± 2.09	13.98 ± 1.76	141.22^***^	P1 < P2 < P3
persistence	9.43 ± 2.58	11.69 ± 2.08	13.13 ± 2.49	208.68^***^	P1 < P2 < P3
stress resistance	8.11 ± 2.84	7.93 ± 2.84	10.69 ± 3.50	91.00^***^	P1 = P2 < P3
optimism	9.40 ± 2.37	11.96 ± 2.02	14.31 ± 1.34	459.16^***^	P1 < P2 < P3
emotional control	9.51 ± 2.52	10.15 ± 2.74	12.52 ± 2.65	107.74^***^	P1 < P2 < P3
empathy	8.92 ± 2.10	10.76 ± 1.91	12.83 ± 2.03	294.94^***^	P1 < P2 < P3
trust	8.61 ± 2.03	10.54 ± 1.93	12.92 ± 2.30	339.40^***^	P1 < P2 < P3
cooperation	9.91 ± 1.81	12.20 ± 1.41	14.30 ± 1.14	681.99^***^	P1 < P2 < P3
tolerance	9.70 ± 2.07	11.87 ± 1.72	14.04 ± 1.53	452.35^***^	P1 < P2 < P3
curiosity	9.30 ± 1.80	11.96 ± 1.53	14.23 ± 1.29	773.02^***^	P1 < P2 < P3
creativity	9.34 ± 1.56	11.28 ± 1.72	13.55 ± 1.59	487.61^***^	P1 < P2 < P3
sociability	8.25 ± 1.89	10.36 ± 2.01	13.41 ± 1.85	527.26^***^	P1 < P2 < P3
assertiveness	7.29 ± 2.37	8.72 ± 2.61	10.58 ± 3.25	113.05^***^	P1 < P2 < P3
energy	8.27 ± 1.91	9.50 ± 2.09	11.92 ± 2.57	221.64^***^	P1 < P2 < P3

^***^ *p*<0.001.

### 3.4 Comparison of online social experiences among the three profiles of socio-emotional skills

A one-way ANOVA was conducted to examine the relationship between the three socio-emotional skills profiles and online social experiences based on the results of the latent profile analysis. For online social positivity, significant differences were found among the three profiles (*F*_(2, 1419)_ = 61.37, *p* < 0.001, *η^2^* = 0.08). *Post hoc* tests indicated that Profile 1 had the lowest scores (*M* = 22.74, *SD* = 7.77), followed by Profile 2 (*M* = 25.95, *SD* = 7.67), while Profile 3 exhibited the highest scores (*M* = 29.88, *SD* = 8.75), with all comparisons being statistically significant (*p* < 0.001). Similarly, significant differences were observed among the three profiles in terms of online social negativity (*F*_(2, 1419)_ = 49.46, *p* < 0.001, *η^2^* = 0.07). However, *post hoc* tests revealed that the pattern of scores for online social negativity was reversed (*p* < 0.001), with scores increasing in the order of Profile 3 (*M* = 14.00, *SD* = 7.18), Profile 2 (*M* = 16.42, *SD* = 5.95), and Profile 1 (*M* = 19.09, *SD* = 6.50). In addition to one-way ANOVA, the BCH method was employed to validate the findings. As shown in **Table 3**, the findings from the BCH method were consistent with those obtained from the one-way ANOVA.

**Table 3:** Comparison of online social experiences among the three latent profiles.

		Between-group variation			
Variable	Type	P1	P2	P3	Overall chi-square test
online social positivity	P1	0			112.06^***^
	P2	38.66^***^	0		
	P3	111.05^***^	39.93^***^	0	
online social negativity	P1	0			86.33^***^
	P2	40.57^***^	0		
	P3	82.86^***^	22.46^***^	0	

^***^ *p*<0.001.

## 4 Discussion

Based on the uses and gratifications theory, the present study employed network analysis and latent profile analysis to explore the relationship between socio-emotional skills and online social experiences and identified key dimensions to support the development of intervention strategies.

### 4.1 The Association between overall socio-emotional skills and online social experiences

The results of this study demonstrated a clear association between overall socio-emotional skills and online social experiences. The network analysis revealed that 9 out of the 15 sub-dimensions of socio-emotional skills were directly related to online social experiences, collectively explaining approximately 20% of the variance in both positive and negative online interactions. Moreover, socio-emotional skills related to online social experiences were positively associated with positive online interactions, while they were primarily negatively associated with negative online experiences. These findings are consistent with previous studies, indicating that socio-emotional skills could modify online social experiences. Casale et al. (47) suggested that individuals with different levels of socio-emotional skills tend to have varying preferences in social media use, which may lead to different types of online social experiences. In addition, Bottaro and Faraci (48) argued that developing socio-emotional skills could help prevent maladaptive social media use, thereby reducing the opportunities for negative online experiences and increasing the chances of positive ones. Similarly, Marín-López et al. (49) found that individuals with higher levels of socio-emotional competence are better able to build positive interpersonal relationships on social media, thus receiving positive online experiences and avoiding negative online experiences. These conclusions were further supported by the findings of the latent profile analysis.

The latent profile analysis identified three distinct patterns of socio-emotional skills among Chinese young adults, characterized as low, moderate, and high socio-emotional skill groups. These profiles reflected meaningful variations in the overall levels of socio-emotional competence. This classification is consistent with previous research (50, 51), which has repeatedly reported heterogeneity socio-emotional skills, often emerging in the form of high versus low in socio-emotional skills. Despite differences in theoretical frameworks and measurement tools across studies, the recurring presence of such stratified profiles suggests a common structural pattern in socio-emotional skills across different populations and cultural contexts. Notably, within each profile in the present study, the relative levels across the 15 sub-dimensions of socio-emotional skills remained largely consistent, indicating a high degree of internal synchrony or coordination among these competencies. This pattern implies that socio-emotional skills are likely to develop and function in an interconnected manner, rather than operating in isolation (52). The results of the network analysis also revealed complex interrelations among the sub-dimensions of socio-emotional skills, suggesting potential synergistic effects among these competencies.

Furthermore, significant differences in online social experiences were observed across the three socio-emotional skill profiles. Specifically, individuals in the high-skill group reported the highest levels of online social positivity, followed by those in the moderate group, while the low-skill group scored the lowest. In contrast, the pattern for online social negativity was reversed, with the highest levels reported by the low-skill group, followed by the moderate group, and the lowest levels observed in the high-skill group. These findings provided additional evidence for the influential role of socio-emotional skills in modifying online social experience. They suggested that individuals with higher socio-emotional skills are more likely to engage in positive online interactions and less likely to experience negative online encounters. According to social cognitive theory, individuals with greater socio-emotional skills are better equipped to interpret social cues and manage interpersonal challenges (53). These abilities are particularly important in the online context, where nonverbal signals are limited and miscommunication is more likely (54). Higher levels of socio-emotional skills may thus help individuals respond more constructively to ambiguous or negative online interactions, reducing conflict and enhancing positive experiences.

### 4.2 The role of socio-emotional skill sub-dimensions in online social experiences

Despite the results suggesting a high degree of coordination among socio-emotional skill sub-dimensions, understanding their individual contributions is essential for identifying specific strengths that influence online social experiences. Such an analysis may offer valuable insights for targeted interventions.

Network analysis revealed that all sub-dimensions within the domain of emotion regulation (stress resistance, optimism, emotional control) were negatively associated with negative online social experiences. Emotion regulation is widely regarded as a core component of socio-emotional skills (55, 56). According to the emotion regulation model proposed by Gross (57), individuals engage in two key processes before emotional responses occur: cognitive change and response modulation. Socio-emotional competencies are likely to contribute meaningfully at both stages. In the cognitive change phase, individuals reinterpret events to alter their emotional impact, a process known as cognitive reappraisal. Baumgartner et al. (58) found that individuals with an optimistic disposition tend to engage more frequently in cognitive reappraisal. In the response modulation phase, individuals manage their emotional expressions, a process that requires a certain degree of emotional control (59). Additionally, stress resilience has been shown to be positively associated with cognitive reappraisal and negatively associated with expressive suppression, thereby enabling individuals to adopt contextually appropriate emotional expression strategies (60). Higher levels of emotion regulation are also associated with greater psychological well-being (61). These findings suggest that enhancing socio-emotional skills, particularly those related to emotion regulation, may help young adults reduce negative emotional experiences and experience more positive emotions in online settings.

Notably, stress resilience emerged as the most central variable within the socio-emotional skill network, as indicated by its highest centrality index. This finding suggests that it occupies a pivotal position in socio-emotional skills. Similarly, the latent profile analysis revealed that individuals in the high socio-emotional skill group exhibited significantly greater stress resilience compared to those in the moderate and low types. These results imply that stress resilience may serve as a key facilitator in socio-emotional competencies at a higher level. Stress resilience may function to stabilize and amplify the existing capacities, enabling individuals to operate at a higher level of socio-emotional skill. When stress resilience reaches a sufficient level, the overall coordination and effectiveness of socio-emotional skills can be optimized. Currently, most researchers conceptualize stress resilience as a dynamic process involving the interaction between risk and protective factors when individuals encounter adversity (62). Rather than being an absolute trait, stress resilience is considered a capacity that can be shaped and enhanced through experience and development, mirroring the modifiability of socio-emotional skills (63). The presence of stress resilience does not guarantee successful coping under all conditions; its effectiveness varies across individuals and contexts. According to the risk-protective model proposed by Garmezy et al. (64), individuals with higher levels of stress resilience are more capable of mobilizing protective factors to buffer the impact of stress, either by maintaining stability or by adapting responsively to challenges. Within the context of the present study, it is plausible that individuals with higher stress resilience are better able to activate and leverage other sub-dimensions of socio-emotional skills, such as emotional control, responsibility, or self-control when navigating complex online social environments. This capacity to coordinate multiple skills under pressure may contribute to their more favorable online social experiences (65).

Additionally, the study found that individuals with higher levels of empathy and sociability are more likely to experience positive online social interactions. Meanwhile, self-control was identified as a potential factor for recognizing individuals who may be more vulnerable to negative online social experiences. These findings provide valuable insights for developing targeted interventions aimed at enhancing positive online interactions and mitigating the impact of negative experiences.

### 4.3 The relationship between positive and negative online social experiences

The finding that positive and negative online social experiences were positively correlated suggests that these experiences may not lie at opposite ends of a single continuum, but rather represent two distinct and co-occurring dimensions of online interaction. In other words, individuals may experience a high level of online social positivity while also encountering a high level of online social negativity. Wu and Yao (66) provided neuroimaging evidence showing that positive and negative experiences activate different neural systems, further reinforcing the conceptual independence of the two constructs. This dual-experience perspective highlights the complexity of digital social environments, where individuals can simultaneously receive emotional support and face interpersonal challenges (9, 14). Moreover, some studies suggested that even positive online social experiences may carry potential risks. For instance, excessive reliance on social support obtained through social media has been linked to problematic smartphone use, which may lead to psychological distress and ultimately contribute to negative online experiences (67). This implies that in digital settings, seemingly beneficial interactions may coexist with or even give rise to adverse outcomes, underscoring the need for a more nuanced understanding of online social dynamics.

### 4.4 Implications

The results of this study highlighted the urgent need to foster socio-emotional skills in young people in the context of widespread social media use. The association between overall socio-emotional skills and online social experiences suggests that interventions aimed at improving young people’s socio-emotional skills should not focus on isolated dimensions. Instead, they should address a comprehensive range of skills that collectively enhance their ability to navigate and engage in meaningful online interactions (68). However, when time and resources are limited, focusing on young people’s stress resilience may be a more efficient approach. Stress resilience plays a dynamic mediating role between protective and risk factors (69). Designing targeted interventions based on this skill may have broader effects on other dimensions of socio-emotional skills, thereby enhancing individuals’ overall socio-emotional competence and, in turn, improving their online social experiences.

Moreover, the distinction between positive and negative online social experiences implied that intervention strategies should be tailored accordingly, rather than adopting a one-size-fits-all approach. While fostering socio-emotional skills such as empathy and sociability may enhance individuals’ capacity to seek and maintain positive social interactions online, addressing negative experiences, such as social rejection or digital embarrassment, may require a greater focus on optimism and emotional regulation. Recognizing these differential pathways is crucial for developing more precise and effective programs that not only promote supportive online environments but also protect individuals from the psychological harm of negative online interactions.

### 4.5 Limitations and future directions

Several limitations should be acknowledged. First, the sample comprised university students from China, which may limit the generalizability of the findings to other populations. Future research should explore whether these patterns hold across diverse groups. Second, both social-emotional skills and online social experiences were assessed using self-report questionnaires, which may introduce biases, such as social desirability effects. To improve the validity of the findings, future studies could incorporate evaluations from parents, teachers, or peers, as well as physiological measures. Lastly, this study employed a cross-sectional design, which prevents us from determining the directionality of the relationship between social-emotional skills and online social experiences. For example, Hatamleh et al. (70) found that positive online experiences can also enhance empathy levels. Therefore, longitudinal studies are needed to explore the causal pathways between these variables more thoroughly.

## 5 Conclusion

This study found a significant association between socio-emotional skills and online social experiences. Individuals with higher levels of socio-emotional skills were more likely to have positive online social experiences, while those with lower levels were more susceptible to negative online social experiences. Among the different dimensions, stress resilience emerged as a key factor in further enhancing the overall level of socio-emotional skills. Additionally, positive and negative online social experiences were found to be independent of each other, implying that interventions should consider both dimensions separately.

## Data Availability

The data analyzed in this study is subject to the following licenses/restrictions: The dataset is not accessible to the public, as it contains information that could potentially violate the privacy of the participants. The data supporting the conclusions of this article can be obtained from the corresponding author. Requests to access these datasets should be directed to ZC, cuiziqian1016@163.com.

## References

[1] Statista. Number of social media users worldwide from 2018 to 2027 (2024). Available online at: https://www.statista.com/statistics/278414/number-of-worldwide-social-network-users/ (Accessed July 19, 2024).

[2] Pew Research Center. Social media use in 2021 (2021). Available online at: https://www.pewresearch.org/internet/2021/04/07/social-media-use-in-2021/ (Accessed April 7, 2021).

[3] Zhang YJ Al Imran Yasin M Alsagoff SABS Hoon AL. The mediating role of new media engagement in this digital age. Front Public Health. (2022) 10:879530. doi: 10.3389/fpubh.2022.879530 35586011 PMC9108457

[4] Vriens E van Ingen E. Does the rise of the Internet bring erosion of strong ties? Analyses of social media use and changes in core discussion networks. New Media Soc. (2018) 20:2432–49. doi: 10.1177/1461444817724169 PMC6256723 30581363

[5] Shensa A Sidani JE Escobar-Viera CG Switzer GE Primack BA Choukas-Bradley S. Emotional support from social media and face-to-face relationships: Associations with depression risk among young adults. J Affect Disord. (2020) 260:38–44. doi: 10.1016/j.jad.2019.08.092 31493637 PMC7383439

[6] Waytz A Gray K. Does online technology make us more or less sociable? A preliminary review and call for research. Perspect Psychol Sci. (2018) 13:473–91. doi: 10.1177/1745691617746509 29758166

[7] Singleton A Abeles P Smith IC. Online social networking and psychological experiences: The perceptions of young people with mental health difficulties. Comput Hum Behavior. (2016) 61:394–403. doi: 10.1016/j.chb.2016.03.011

[8] Clark JL Algoe SB Green MC. Social network sites and well-being: The role of social connection. Curr Dir psychol Science. (2017) 27:32–7. doi: 10.1177/0963721417730833

[9] Kent de Grey RG Uchino BN Baucom BR Smith TW Holton AE Diener EF. Enemies and friends in high-tech places: the development and validation of the Online Social Experiences Measure. Digital Health. (2019) 5:1–20. doi: 10.1177/2055207619878351 PMC6759713 31579526

[10] Paceley MS Goffnett J Sanders L Gadd-Nelson J. Sometimes you get married on Facebook: The use of social media among nonmetropolitan sexual and gender minority youth. J Homosex. (2022) 69:41–60. doi: 10.1080/00918369.2020.1813508 32875962

[11] Chadwick DD Fullwood C. An online life like any other: Identity, self-determination, and social networking among adults with intellectual disabilities. Cyberpsychol Behav Soc Netw. (2018) 21:56–64. doi: 10.1089/cyber.2016.0689 28846024

[12] Magis-Weinberg L Gys CL Berger EL Domoff SE Dahl RE. Positive and negative online experiences and loneliness in Peruvian adolescents during the COVID-19 lockdown. J Res Adolesc. (2021) 31:717–33. doi: 10.1111/jora.12666 PMC8646854 34448303

[13] Cole DA Nick EA Zelkowitz RL Roeder KM Spinelli T. Online social support for young people: Does it recapitulate in-person social support; can it help? Comput Hum Behav. (2017) 68:456–64. doi: 10.1016/j.chb.2016.11.058 PMC5630180 28993715

[14] Uchino BN Jordan KD Smith TW. Positive and negative online social experiences and self-rated health: Associations and examination of potential pathways. Health Psychol. (2024) 43:125–31. doi: 10.1037/hea0001338 38032612

[15] Shi YY Luo YLL Liu YZ Yang ZY. Affective experience on social networking sites predicts psychological well-being off-line. Psychol Rep. (2019) 122:1666–77. doi: 10.1177/0033294118789039 30080110

[16] Prizeman K Weinstein N McCabe C. Effects of mental health stigma on loneliness, social isolation, and relationships in young people with depression symptoms. BMC Psychiatry. (2023) 23:527. doi: 10.1186/s12888-023-04991-7 37479975 PMC10362624

[17] Stevens F Nurse JRC Arief B. Cyber stalking, cyber harassment, and adult mental health: A systematic review. Cyberpsychol Behav Soc Netw. (2021) 24:367–76. doi: 10.1089/cyber.2020.0253 33181026

[18] Kee DMH Anwar A Vranjes I. Cyberbullying victimization and suicide ideation: The mediating role of psychological distress among Malaysian youth. Comput Hum Behavior. (2024) 150:108000. doi: 10.1016/j.chb.2023.108000

[19] Pfetsch JS Schultze-Krumbholz A Lietz K. Can acting out online improve adolescents’ well-being during contact restrictions? A first insight into the dysfunctional role of cyberbullying and the need to belong in well-being during COVID-19 pandemic-related contact restrictions. Front Psychol. (2022) 12:787449. doi: 10.3389/fpsyg.2021.787449 35082725 PMC8784371

[20] Bonsaksen T Steigen AM Stea TH Kleppang AL Lien L Leonhardt M. Negative social media-related experiences and lower general self-efficacy are associated with depressive symptoms in adolescents. Front Public Health. (2023) 10:1037375. doi: 10.3389/fpubh.2022.1037375 36684882 PMC9853181

[21] Garcia MA Cervantes A Rodriguez-Crespo A Drozdova AD Cooper TV. Online social experiences among Hispanic emerging adults: Associations with mental and sleep health. Psychol Popular Media. (2024). doi: 10.1037/ppm0000564

[22] Katz E Blumler JG Gurevitch M. Uses and gratifications research. Public Opin Quarterly. (1973) 37:509–23. doi: 10.1086/268109

[23] Chao M Xue DN Liu T Yang HB Hall BJ. Media use and acute psychological outcomes during COVID-19 outbreak in China. J Anxiety Disord. (2020) 74:102248. doi: 10.1016/j.janxdis.2020.102248 32505918 PMC7255752

[24] Brown RM Roberts SG Pollet TV. HEXACO personality factors and their associations with Facebook use and Facebook network characteristics. Psychol Rep. (2025) 128:1942–66. doi: 10.1177/00332941231176403 PMC11977835 37235982

[25] Chua YP Chua YP. Do computer-mediated communication skill, knowledge and motivation mediate the relationships between personality traits and attitude toward Facebook? Comput Hum Behav. (2017) 70:51–9. doi: 10.1016/j.chb.2016.12.034

[26] Evangelou SM Michanetzi EL Xenos MN. Exploring the impact of negative online feedback on well-being: A comprehensive analysis incorporating Big-Five personality traits and physiological responses. Comput Hum Behav Reports. (2024) 15:100457. doi: 10.1016/j.chbr.2024.100457

[27] Gosling SD Augustine AA Vazire S Holtzman N Gaddis S. Manifestations of personality in online social networks: Self-reported Facebook-related behaviors and observable profile information. Cyberpsychol Behav Soc Netw. (2011) 14:483–8. doi: 10.1089/cyber.2010.0087 PMC3180765 21254929

[28] Chow TS Wan HY. Is there any ‘Facebook depression’? Exploring the moderating roles of neuroticism, Facebook social comparison and envy. Pers Individ Differences. (2017) 119:277–82. doi: 10.1016/j.paid.2017.07.032

[29] Goldberg LR. The structure of phenotypic personality traits. Am Psychol. (1993) 48:26–34. doi: 10.1037//0003-066x.48.1.26 8427480

[30] Terracciano A McCrae RR Costa PT Jr. Intra-individual change in personality stability and age. J Res Pers. (2010) 44:31–7. doi: 10.1016/j.jrp.2009.09.006 PMC2839250 20305728

[31] Jagers RJ Rivas-Drake D Williams B. Transformative social and emotional learning (SEL): Toward SEL in service of educational equity and excellence. Educ Psychol. (2019) 54:162–84. doi: 10.1080/00461520.2019.1623032

[32] Soto CJ Napolitano CM Sewell MN Yoon HJ Roberts BW. Going beyond traits: Social, emotional, and behavioral skills matter for adolescents’ success. Soc psychol Pers Science. (2022) 15:33–45. doi: 10.1177/19485506221127483

[33] Soto CJ Napolitano CM Sewell MN Yoon HJ Roberts BW. An integrative framework for conceptualizing and assessing social, emotional, and behavioral skills: The BESSI. J Pers Soc Psychol. (2022) 123:192–222. doi: 10.1037/pspp0000401 35113631

[34] Wang FM King RB. Developing the short form of the survey on social and emotional skills (SSES-SF). J Pers Assessment. (2024). doi: 10.1080/00223891.2024.2416416 39540658

[35] Jones SM McGarrah MW Kahn J. Social and emotional learning: A principled science of human development in context. Educ Psychol. (2019) 54:129–43. doi: 10.1080/00461520.2019.1625776

[36] Epskamp S Borsboom D Fried EI. Estimating psychological networks and their accuracy: A tutorial paper. Behav Res Methods. (2018) 50:195–212. doi: 10.3758/s13428-017-0862-1 28342071 PMC5809547

[37] OECD. Social and emotional skills for better lives: findings from the OECD survey on social and emotional skills 2023. Paris: OECD Publishing (2024).

[38] Hofmans J Wille B Schreurs B. Person-centered methods in vocational research. J Vocational Behavior. (2020) 118:103398. doi: 10.1016/j.jvb.2020.103398

[39] Constantin MA Schuurman NK Vermunt JK. A general Monte Carlo method for sample size analysis in the context of network models. Psychol Methods. (2023). doi: 10.1037/met0000555 37428726

[40] Hu L Bentler PM. Fit indices in covariance structure modeling: Sensitivity to underparameterized model misspecification. psychol Methods. (1998) 3:424–53. doi: 10.1037/1082-989X.3.4.424

[41] Chen F Curran PJ Bollen KA Kirby J Paxton P. An empirical evaluation of the use of fixed cutoff points in RMSEA test statistic in structural equation models. Sociol Methods Res. (2008) 36:462–94. doi: 10.1177/0049124108314720 PMC2743032 19756246

[42] R Core Team. R: A Language and Environment for Statistical Computing (2024). Available online at: https://www.R-project.org/.

[43] Opsahl T Agneessens F Skvoretz J. Node centrality in weighted networks: Generalizing degree and shortest paths. Soc Networks. (2010) 32:245–51. doi: 10.1016/j.socnet.2010.03.006

[44] Muthén LK Muthén BO. Mplus: Statistical Analysis With Latent Variables. Los Angeles, CA (2019).

[45] Bolck A Croon M Hagenaars J. Estimating latent structure models with categorical variables: One-step versus three-step estimators. Political Analysis. (2004) 12:3–27. doi: 10.1093/pan/mph001

[46] Cheung T Jin Y Lam S Su Z Hall BJ Xiang YT. Network analysis of depressive symptoms in Hong Kong residents during the COVID-19 pandemic. Transl Psychiatry. (2021) 11:460. doi: 10.1038/s41398-021-01543-z 34489416 PMC8419676

[47] Casale S Tella L Fioravanti G. Preference for online social interactions among young people: Direct and indirect effects of emotional intelligence. Pers Individ Differences. (2013) 54:524–9. doi: 10.1016/j.paid.2012.10.023

[48] Bottaro R Faraci P. The use of social networking sites and its impact on adolescents’ emotional well-being: A scoping review. Curr Addict Rep. (2022) 9:518–39. doi: 10.1007/s40429-022-00445-4 PMC9516496 36185594

[49] Marín-López I Zych I Ortega-Ruiz R Hunter SC Llorent VJ. Relations among online emotional content use, social and emotional competencies and cyberbullying. Children Youth Serv Review. (2020) 108:104647. doi: 10.1016/j.childyouth.2019.104647

[50] Jiang Y Zhang LJ Chen C. Latent profiles of Chinese students’ social-emotional learning competencies and their associations with academic motivation and achievement. Learn Individ Differences. (2024) 116:102580. doi: 10.1016/j.lindif.2024.102580

[51] Tan K Sinha G Shin OJ Wang Y. Patterns of social-emotional learning needs among high school freshmen students. Children Youth Serv Review. (2018) 86:217–25. doi: 10.1016/j.childyouth.2018.01.033

[52] Zins JE Weissberg RP Wang MC Walberg HJ. Building Academic Success on Social and Emotional Learning: What Does the Research Say? New York: Teachers College Press (2004).

[53] McKown C Gumbiner LM Russo NM Lipton M. Social-emotional learning skill, self-regulation, and social competence in typically developing and clinic-referred children. J Clin Child Adolesc Psychol. (2009) 38:858–71. doi: 10.1080/15374410903258934 20183669

[54] Kelly L Miller-Ott AE. Perceived miscommunication in friends’ and romantic partners’ texted conversations. South Communication J. (2018) 83:267–80. doi: 10.1080/1041794X.2018.1488271

[55] Kao K Tuladhar CT Tarullo AR. Parental and family-level sociocontextual correlates of emergent emotion regulation: Implications for early social competence. J Child Fam Stud. (2020) 29:1630–41. doi: 10.1007/s10826-020-01706-4 PMC8323769 34334997

[56] Blair BL Perry NB O’Brien M Calkins SD Keane SP Shanahan L. Identifying developmental cascades among differentiated dimensions of social competence and emotion regulation. Dev Psychol. (2015) 51:1062–73. doi: 10.1037/a0039472 PMC4520405 26147773

[57] Gross JJ. The emerging field of emotion regulation: An integrative review. Rev Gen Psychol. (1998) 2:271–99. doi: 10.1037/1089-2680.2.3.271

[58] Baumgartner JN Schneider TR Capiola A. Investigating the relationship between optimism and stress responses: A biopsychosocial perspective. Pers Individ Differences. (2018) 129:114–8. doi: 10.1016/j.paid.2018.03.021

[59] Seixas R Pignault A Houssemand C. Emotion regulation questionnaire-adapted and individual differences in emotion regulation. Eur J Psychol. (2021) 17:70–84. doi: 10.5964/ejop.2755 33737975 PMC7957848

[60] Rodríguez-Rey R Guerra Corral M Collazo-Castiñeira P Collado S Caro-Carretero R Cantizano A. Predictors of mental health in healthcare workers during the COVID-19 pandemic: The role of experiential avoidance, emotion regulation and resilience. J Adv Nurs. (2024) 80:4089–102. doi: 10.1111/jan.16122 38382909

[61] Rueth J-E Lohaus A. Process-oriented measurement of emotion regulation: General and specific associations with psychosocial adjustment and well-being in (pre-)adolescence. Front Psychiatry. (2022) 13:904389. doi: 10.3389/fpsyt.2022.904389 35815049 PMC9259935

[62] Rutter M. Resilience concepts and findings: Implications for family therapy. J Family Ther. (1999) 21:119–44. doi: 10.1111/1467-6427.00108

[63] Crane MF Searle BJ Kangas M Nwiran Y. How resilience is strengthened by exposure to stressors: the systematic self-reflection model of resilience strengthening. Anxiety Stress Coping. (2019) 32:1–17. doi: 10.1080/10615806.2018.1506640 30067067

[64] Garmezy N Masten AS Tellegen A. The study of stress and competence in children: a building block for developmental psychopathology. Child Dev. (1984) 55:97–111. doi: 10.2307/1129837 6705637

[65] Bermes A Gromek CL. Don’t want it anymore? Resilience as a shield against social media-induced overloads. In: Ahlemann F Schütte R Stieglitz S, editors. Innovation Through Information Systems: Volume II: A Collection of Latest Research on Technology Issues. Springer, Cham (2021). p. 451–8.

[66] Wu DX Yao SQ. Theoretic framework of neural methods of positive/negative emotional parallel. Chin J Clin Psychol. (2007) 15:493–5. doi: 10.16128/j.cnki.1005-3611.2007.05.022

[67] Ma YJ Zhou ZY Ye CX Liu MX. Online social support and problematic Internet Use-a meta-analysis. Addict Behav. (2025) 160:108160. doi: 10.1016/j.addbeh.2024.108160 39265417

[68] Elias MJ Zins JE Weissberg RP. Promoting Social and Emotional Learning: Guidelines for Educators. New York: ASCD Press (1997).

[69] Richardson GE. The metatheory of resilience and resiliency. J Clin Psychol. (2002) 58:307–21. doi: 10.1002/jclp.10020 11836712

[70] Hatamleh IHM Safori AO Habes M Tahat O Ahmad AK Abdallah RA-Q. Trust in social media: Enhancing social relationships. Soc Sci. (2023) 12:416. doi: 10.3390/socsci12070416

